# Twenty-Ninth Annual Commencement of the Buffalo Medical College

**Published:** 1875-02

**Authors:** 


### Editorial.

### Twenty-Ninth Annual Commencement of the Buffalo Medical College.

The twenty-ninth Annual Commencement exercises of the Medical Department of the University of Buffalo, were held at St. James Hall, Tuesday evening, February 23d.

The exercises were opened with prayer by Rev. G. W. Heacock, after which the Dean, Prof. Potter, read the report of the proceedings of the Council as follows:

The Council of the University of Buffalo, at a meeting held in this city today, conferred the honorary degree of Doctor of Medicine upon W. McCollum, of Lockport, Niagara county, N. Y. They also, in accordance with the Faculty and Curators of the Medical Department, directed that the degree of Doctor in Medicine be conferred on each of the following gentlemen:

Charles Cary, Buffalo; Jacob Chambers, Jr., Kingston, N. Y.; Henry Franklin Fullerton, Buffalo; Albert Gale Leland, Trumbull, Ohio; William Penn Clothier, Buffalo; Harmon Judson Ashley, Sandusky, N. Y.; William Lewis Allen, Hoyt's Corners, N. Y.; Arthur John Lawrence, Rochester, N. Y.; William Evans Hughes, Aylmer, Ont.; Joel Henry Greene, Franklinville, N. Y.; Philip Alexis McCrea, Portville, N. Y.; Zera J. Lusk, Clarence, N. Y.; John Archibald McDonell, Barton, N. Y.; Fred Francis Webster, East Bloomfield, N. Y.; Frederick DeForest Lamb, Castle Creek, N. Y.; Otis Augustus Jackway, Breesport, N. Y.; Fred C. Beals, East Randolph, N. Y.; Dudley S. Brainard, Rosendale, Wis.; Frederick Austin Manderville, Rochester, N. Y.; Charles Alfred Booth, Brooklyn, N. Y.; William Harvey DeKay, Forrestburg, N. Y.; George Daniel Bradford, Cortland, N. Y.; James Henry Budd, North Hector, N. Y.; Wallace Sibley, Ischua, N. Y.; Frank Adelbert Dutton,

Eagle, N. Y.; George Launcelot Taylor, East Hamburg, N. Y.; David T. Pierce, Cambridge, N. Y.; La Vega Rathbun, Howard, N. Y.; Arthur DeVoe, Mercer, Pa.; Le Verne Augustine Badger, Sheffield, Pa.; Edward Girard Brown, Port Allegany, Pa.; Samuel Griswold Dorr, Dansville, N. Y.; Edwin Eugene Whitcomb, Albion, N. Y.; John Pitts Colgrove, Allegany, N. Y.; Charles Herbert Davie, Buckfield; Alexander Ross Sutherland, Tonawanda, N. Y.; Frederick Kiehbiel, Clarence, N. Y.; John Daniel Maloy, Buffalo, Charles Munroe Jones, Charlotte, N. Y.; John Harrison Clark, Wyoming, N. Y.; George Benjamin Bishop, Silver Creek, N. Y.; Charles Milton Garlock, Lindenville, N. Y.; Frank W. Miller, Whippany, N. J.; Caspian R. Morrow, Buffalo.

And the council also at the same meeting directed that the degree of doctor in medicine be conferred upon Charles Osman Chester, at our next commencement, when he shall have attained his majority.

The following Theses were deemed by the faculty and curators worthy of honorable mention:

1. A Thesis on Diet; by George Launcelot Taylor.
2. A Thesis on Hysterical Progressive Locomotor Ataxia; by Charles Cary.
3. A Thesis on Hydrothorax; by Henry Franklin Fullerton.
4. A Thesis on the Literary Requirements of the Physician and his ability to Control Disease; by Edward Girard Brown.

At the conclusion of the report, the members of the class were called upon the stage and received their diplomas from Hon. O. H. MARSHALL, President of the council, following which came the address of the Graduating Class, by Prof. JAMES P. WHITE.

As the Alumni Association also held its inaugural meeting on this occasion, the committee having the matter in charge, had made arrangements with Dr. W. W. POTTER, of Mount Morris, (class of 59) to deliver an address to the Alumni.

At the conclusion of Prof. WHITE's remarks, Dr. POTTER was introduced and delivered an interesting address. We regret that time and space will not permit us to give a synopsis of these addresses, we shall hope however in our next number to give a more extended report, more particularly of the proceedings of the Alumni Association. The proceedings at the hall were agreeably interspersed by music from Wahle's orchestra.

